# Cryo-EM Structure (4.5-Å) of Yeast Kinesin-5–Microtubule Complex Reveals a Distinct Binding Footprint and Mechanism of Drug Resistance

**DOI:** 10.1016/j.jmb.2019.01.011

**Published:** 2019-02-15

**Authors:** Ottilie von Loeffelholz, Alejandro Peña, Douglas Robert Drummond, Robert Cross, Carolyn Ann Moores

**Affiliations:** 1 Institute of Structural and Molecular Biology, Birkbeck College, London, WC1E 7HX, UK; 2 Division of Biomedical Cell Biology, Warwick Medical School, Coventry, CV4 7AL, UK

**Keywords:** AMPPNP, adenosine 5′-(β,γ-imido)triphosphate, CNB, cover neck bundle, cryo-EM, cryo-electron microscopy, Cut7MD, Cut7 motor domain, Mam_tub, mammalian brain tubulin, MT, microtubule, Sp_tub, *S. pombe* tubulin, STLC, (+)-*S*-trityl-l-cysteine, cytoskeleton, motor, mitosis, Cut7, 3D reconstruction

## Abstract

Kinesin-5s are microtubule-dependent motors that drive spindle pole separation during mitosis. We used cryo-electron microscopy to determine the 4.5-Å resolution structure of the motor domain of the fission yeast kinesin-5 Cut7 bound to fission yeast microtubules and explored the topology of the motor–microtubule interface and the susceptibility of the complex to drug binding. Despite their non-canonical architecture and mechanochemistry, *Schizosaccharomyces pombe* microtubules were stabilized by epothilone at the taxane binding pocket. The overall Cut7 footprint on the *S. pombe* microtubule surface is altered compared to mammalian tubulin microtubules because of their different polymer architectures. However, the core motor–microtubule interaction is tightly conserved, reflected in similar Cut7 ATPase activities on each microtubule type. AMPPNP-bound Cut7 adopts a kinesin-conserved ATP-like conformation including cover neck bundle formation. However, the Cut7 ATPase is not blocked by a mammalian-specific kinesin-5 inhibitor, consistent with the non-conserved sequence and structure of its loop5 insertion.

## Highlights

• Epothilone binds at the taxane binding site to stabilize *S. pombe* microtubules.
• *S. pombe* Cut7 has a distinct binding footprint on *S. pombe* microtubules.
• The core interface driving microtubule activation of motor ATPase is conserved.
• Loop5 of Cut7 adopts a distinctive conformation rendering Cut7 ATPase insensitive to STLC inhibition.

## Graphical abstract

**Figure:**
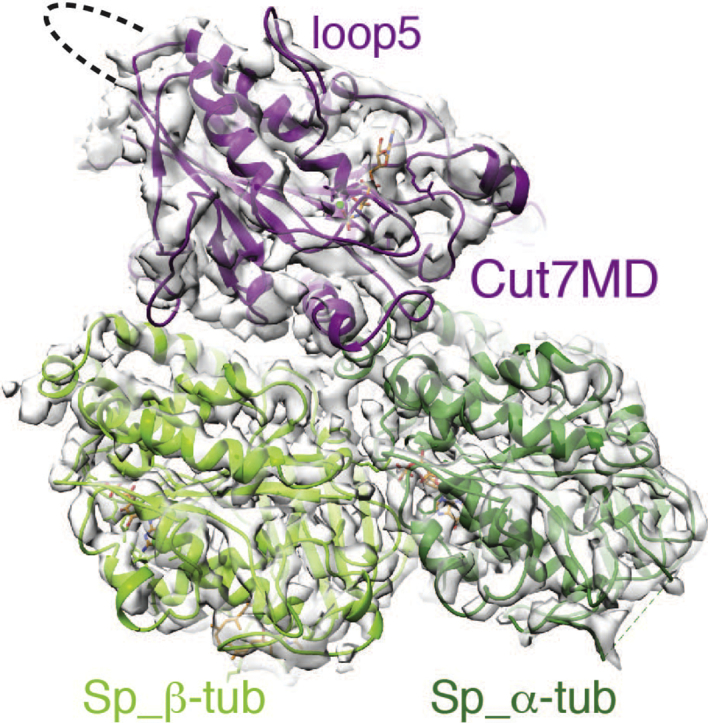

Members of the kinesin superfamily of microtubule (MT)-based, ATP-driven molecular motors play multiple essential roles in cell division, reflecting the dynamic complexity of the machinery required for accurate chromosome segregation. Kinesin-5 motors are conserved among eukaryotes and undertake related roles in cell division, driving spindle pole separation and stabilization of spindle bipolarity [1]. These roles are enabled by the quaternary structure of kinesin-5s, which forms dumbbell shaped antiparallel tetramers that support spindle MT cross linking and sliding. Cut7, the sole kinesin-5 in fission yeast, was among the first mitotic kinesins to be identified and is essential for spindle pole body separation [2]. While most kinesin-5 motors take steps toward MT plus ends [3], several yeast kinesin-5s including Cut7 have been shown to be bidirectional, being able to move to MT plus or minus ends according to molecular context [4], [5], [6], [7], [8]. Although the precise molecular mechanism(s) of direction change in these yeast motors are not known, the capacity for direction change appears to be an emergent property of oligomeric yeast kinesin-5 motor constructs [6], [8]. Understanding of the structure, track interaction and biochemical properties of monomeric yeast kinesin-5s such as Cut7 provides important molecular context for ultimately dissecting such sophisticated motor behavior.

αβ-Tubulin heterodimers are the building blocks of MTs and are among the most highly conserved proteins in eukaryotes. However, the specificity of the kinesin–MT interaction suggests that even subtle differences in the MT track could influence motor function. Tubulin purified from mammalian brain remains the default substrate for investigation of kinesin function. However, it is a heterogeneous mix of multiple tubulin isoforms and post-translationally modified tubulins, so although these sources of diversity influence motor properties [9], their individual contributions to motor function cannot be discriminated. Recent advances in preparation of tubulin from a range of biological sources are highlighting the potential for diverse behavior even among nominally highly conserved tubulins [10], [11], [12], [13], [14], [15], [16]. Purified native fission yeast tubulin contains a mixture of α-tubulin1/2 isoforms (87% sequence identity), a single β-tubulin isoform and no post-translational modifications [11]. We recently showed that the structural properties of fission yeast tubulin (abbreviated here to Sp_tub) are distinct from mammalian brain tubulin (Mam_tub) with respect to the angle of protofilament skew within the MT lattice and the different structural response to tubulin GTPase activity [15]. Furthermore, it is well established that the sensitivity of budding yeast tubulin to MT-binding drugs differs from mammalian tubulin [17], [18].

To explore the important and coevolved facet of motor functionality that is the kinesin–MT interface and exploiting the availability of purified native fission yeast tubulin, we prepared Sp_tub MTs in the presence of the drug epothilone. Using recombinantly expressed Cut7 motor domain (Cut7MD), Sp_tub MTs were bound by Cut7MD in the presence of the non-hydrolyzable ATP analogue adenosine 5′-(β,γ-imido)triphosphate (AMPPNP) and low-dose cryo-electron microscopy (cryo-EM) movies of these complexes were collected. Although epothilone is chemically distinct from taxanes, it binds the taxane binding site in β-tubulin [18], [19]. However, in contrast to taxanes, epothilone stabilizes both Mam_tub MTs and budding yeast MTs [14], [18]. Epothilone was necessarily to vizualize the Sp_tub MTs in our cryo-EM experiments, demonstrating that this drug also stabilizes Sp_tub MTs.

The cryo-EM images reveal the slight PF skew in the Sp_tub MT lattice (Fig. S1A), as previously described [19]. However, the regular, 8-nm-spaced binding characteristic of most kinesin motor domains, including human kinesin-5—for example, Refs [20], [21], [22]—was also observed for Cut7MD (Fig. S1A). This is distinct, however, from the super-stoichiometric binding observed for the budding yeast kinesin-5 Cin8 motor domain [23], which depends on a large insert in loop8 that is not present in Cut7. Thus, while some yeast kinesin-5 motor constructs can exhibit divergent properties, in our cryo-EM experiment, Cut7MD displays a canonical kinesin binding pattern in which it labels every tubulin dimer on Sp_tub MTs. The Cut7MD-bound 13 PF Sp_tub MT data were processed as previously described [15], [24], yielding a reconstruction of 4.5-Å overall resolution (0.143 Fourier Shell Correlation criterion; Fig. S1B–D). This structure of a yeast motor bound to its native MT track is the best-resolved reconstruction of a MT-bound kinesin-5 motor determined to date and allows us to map the motor–MT interface and motor conformation precisely.

In our Sp_tub MT reconstruction, clear density corresponding to epothilone is indeed present in the taxane pocket of β-tubulin that faces the MT lumen (Fig. 1a, b). This shows that epothilone binds Sp_tub similarly to Mam_tub [19] despite the unusual architectures of Sp_tub MTs and sequence divergence in the M-loop region (e.g., Sp_tub S279; Mam_tub Q281). Comparison with our previous drug-free Sp_tub MT structure bound by the protein Mal3, in which the taxane pocket is empty (Fig. 1b, middle) [15], together with difference density calculations (Fig. 1b, bottom) supports the assignment of this density. The drug density in our reconstruction is consistent with the epothilone configuration revealed by X-ray crystallography in complex with Mam_tub dimers [19] and is modeled as such. Determining whether the precise conformation of drug binding is influenced by the polymeric state of tubulin is an important question but will only be possible once cryo-EM MT reconstructions are determined at well below 3-Å resolution where the position of each atom can be determined with high confidence.

**Fig. 1:**
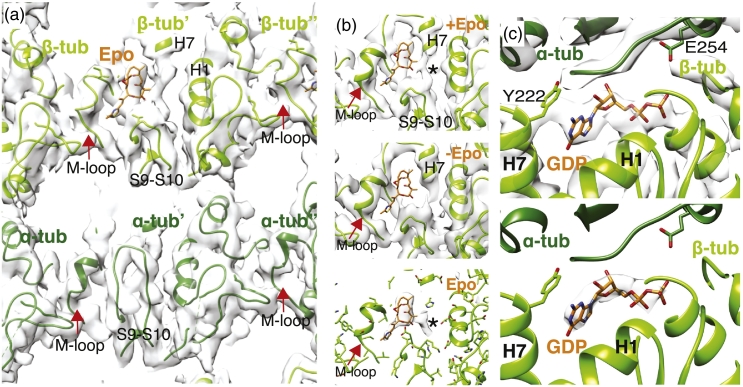
The 4.5-Å resolution reconstruction of *Schizosaccharomyces pombe* Cut7MD-AMPPNP bound to Sp_tub MT shows epothilone bound at the β-tubulin taxane site of Sp_tub MTs. (a) Inter-PF lateral contacts viewed from the MT lumen, highlighting key secondary structure features and bound epothilone (Epo, orange). (b) The taxane binding pocket in β-tubulin where density corresponding to epothilone is visible (top), compared with our previous structure of Sp_tub MTs without epothilone (middle) and the difference density of these two reconstructions ± epothilone (bottom) [15]. An epothilone molecule is docked for comparison in the epothilone density (middle) but lies outside this cryo-EM density. *The top and bottom panels indicate unassigned density that may reflect mobility or alternate conformations of the drug in the pocket. (c) Top, Sp_tub β-tubulin E-site with β-tubulin in light green ribbon and α-tubulin in dark green ribbon, showing density consistent with bound GDP (in sticks); bottom, ribbon depiction of the atomic model of the Sp_tub MT E-site with density corresponding to the bound nucleotide shown in surface representation. This density is the calculated difference between our cryo-EM reconstruction and simulated 4.4-Å resolution density from the atomic models, calculated using Chimera. This supports the conclusion that the E-site nucleotide in Sp_tub MTs is GDP and thus that GTP hydrolysis has occurred in these MTs. *S. pombe* MTs were assembled from tag-free, dual isoform purified endogenous tubulin [11] in PEM buffer [100 mM Pipes–KOH, 1 mM MgSO_4_, 2 mM EGTA, adjusted to pH 6.9 with KOH) mixed 1:1 with Mes polymerization buffer [100 mM Mes (pH 6.5), 1 mM MgCl_2_, 1 mM EGTA, 1 mM DTT). Tubulin (30 μM) was polymerized in the presence of 5 mM GTP together with 25 μM monomeric Mal3 (residues 1–143), expressed in *Escherichia coli* and purified as previously described [15], except that the N-terminal His_6_ purification tag was removed by TEV protease cleavage. Monomeric Mal3 was added to bias the MT population to 13 PF architecture during polymerization in order to facilitate subsequent structure determination, but Mal3 itself is not visible in the final reconstruction, presumably due to dissociation during sample preparation. MTs were polymerized at 32 °C for 1 h. Epothilone B [in DMSO (Stratech UK)] at a final concentration of 50 μM was added in the final 15 min of polymerization. Sp_tub MT (6 μM) was mixed with 100 μM Cut7MD-AMPPNP at room temperature and 4 μl of the mixture was applied immediately onto glow-discharged Quantifoil R 2/2 holey carbon grids, which were blotted and plunge frozen into liquid ethane using a Vitrobot IV (FEI) operating at room temperature and 100% humidity. Movies were collected manually on a 300 kV Tecnai G2 Polara (FEI) microscope equipped with a Quantum energy filter and K2 Summit direct electron detector (Gatan) in counting mode, recording a total of 606 movies with a total dose in each of 30*e*^−^/Å^2^ fractioned into 50 frames at a pixel size of 1.39 Å/px. Initial frame alignment was performed using IMOD [39]. A second local alignment step was performed with Scipion using the optical flow method [40]. In the final reconstruction only frames 2–21 were included resulting in a total dose of 12*e*^−^/Å^2^. A total of 12,543 MT segments were selected in 908-Å^2^ boxes in Boxer [41] using the helix option and choosing an overlap with three tubulin dimers (240 Å) unique in each box. Of the 748 MTs that were initially boxed, 669 MTs with 13_3 architecture were selected. The final 3D reconstruction contained 33,007 segments, reboxed with one unique tubulin dimer per box, and was calculated using a semi-automated single particle approach for pseudo-helical assemblies in SPIDER and FREALIGN [42]. The MT Fourier transform layer lines were used to calculate the average helical repeat distance for the Sp_tub heterodimer. The final reconstruction was automatically *B*-factor sharpened in RELION with an automated calculated *B*-factor of − 234 [43]. As expected from the substoichiometric concentrations present during sample preparation, no density for the Mal3 added during MT polymerization was present in the final reconstruction. The overall resolution of the masked reconstruction was 4.5 Å (0.143 Fourier Shell Correlation). Atomic models were calculated refined (see Table 1) and structures were visualized using Chimera [44].

**Table 1:** Refinement statistics and model geometry for the Cut7/Sp_tub model

Resolution used for refinement	4.5 Å
Molprobity score	1.97
RMSD (bonds, Å)	0.01
RMSD (angles)	0.98
All atoms clashscore	8.35
Ramachandran outliers	0%
Ramachandran allowed	8.98%
Ramachandran favored	91.02%
Rotamer outliers	0.2%

Refinement statistics and model geometry calculated in Phenix [46] and Molprobity [47]. Homology models for the *S. pombe* αβ-tubulin dimer and Cut7MD were adjusted from previous depositions (PDB: 5MJS and 5M5I (with side-chain position information for the Cut7MD from 3HQD), respectively) using Chimera and Coot [48]. The structure of epothilone B was downloaded from the grade web server (http://grade.globalphasing.org) and fitted into the density according to the previously determined high-resolution structure of the tubulin–stathmin–TTL–Epothilone complex [19]. The atomic model was real space refined in Phenix [46] using the EM-map filtered to 4.5 Å. To improve the model geometry, phenix.reduce [49] was also used.

Several recent studies have shown that the mechanochemistry of yeast tubulins is different from that of mammalian tubulins: specifically, the GTPase-dependent MT lattice compaction that is well described in mammalian tubulin [25], [26], [27], [28] either does not occur [15] or is altered in yeast tubulins [14]. Density in the nucleotide site of β-tubulin in our epothilone-bound Sp_tub MTs is consistent with bound GDP (Fig. 1c). Furthermore, the average helical repeat of the Sp_tub heterodimer remained ~ 83 Å in the presence of epothilone: 82.98 ± 0.01 Å (mean and s.d., n = 748, split into three groups), compared to the previously calculated GTP-Sp_tub = 83.26 ± 0.01 Å (mean and s.d.) and Mal3–143 + GTP-Sp_tub = 82.91 ± 0.02 Å (mean and s.d.) [15]. The small differences compared to previous reconstructions are statistically significant [GTP-Sp_tub, *p* < 0.0001 (*t* test); Mal3–143 + GTP-Sp_tub, *p* < 0.006 (*t* test)] but are very small compared to the equivalent difference seen for mammalian MTs [50]. Thus, epothilone binding does not block the Sp_tub GTPase, induce major lattice changes, or cause detectable local differences in its binding pocket (Fig. 1b). However, without the addition of epothilone, these Sp_tub MTs depolymerize. Therefore, epothilone stabilization of yeast MTs is unlikely to depend on effects related to MT lattice compaction/expansion, but rather on other facets of MT structure, for example, the lateral contacts between PFs. We suggest that epothilone binding stabilizes the lateral contacts between adjacent β-tubulins and thereby inhibits MT catastrophe, similar to the Mam_tub MT stabilization mechanism recently proposed for the important chemotherapy drug, paclitaxel [28] (see also Refs. [27], [29], [30]).

The Cut7MD binds to the outside surface of Sp_tub MTs with helix-α4 of Cut7MD centered on the αβ-tubulin intradimer interface (Fig. 2a); this was also seen on Mam_tub MTs at subnanometer resolution [6] but is now visualized at substantially higher resolution (~ 4.5 Å at the binding interface; Fig. S1D). Consistent with sample preparation conditions, there is strong density in the nucleotide binding pocket corresponding to bound Mg-AMPPNP (Fig. 2a, b). The conserved nucleotide-binding loops—the P-loop, loop 9 (containing switch I) and loop 11 (containing switch II) (Fig. S2)—adopt the compact conformation seen in other kinesin motors in the presence of this nucleotide analogue [6], [20], [21], [31], [32]. The observation that the loop11 helical turn is not connected to the MT surface (Fig. 2b, pink arrow) is also consistent with previously characterized ATP-like conformations.

**Fig. 2:**
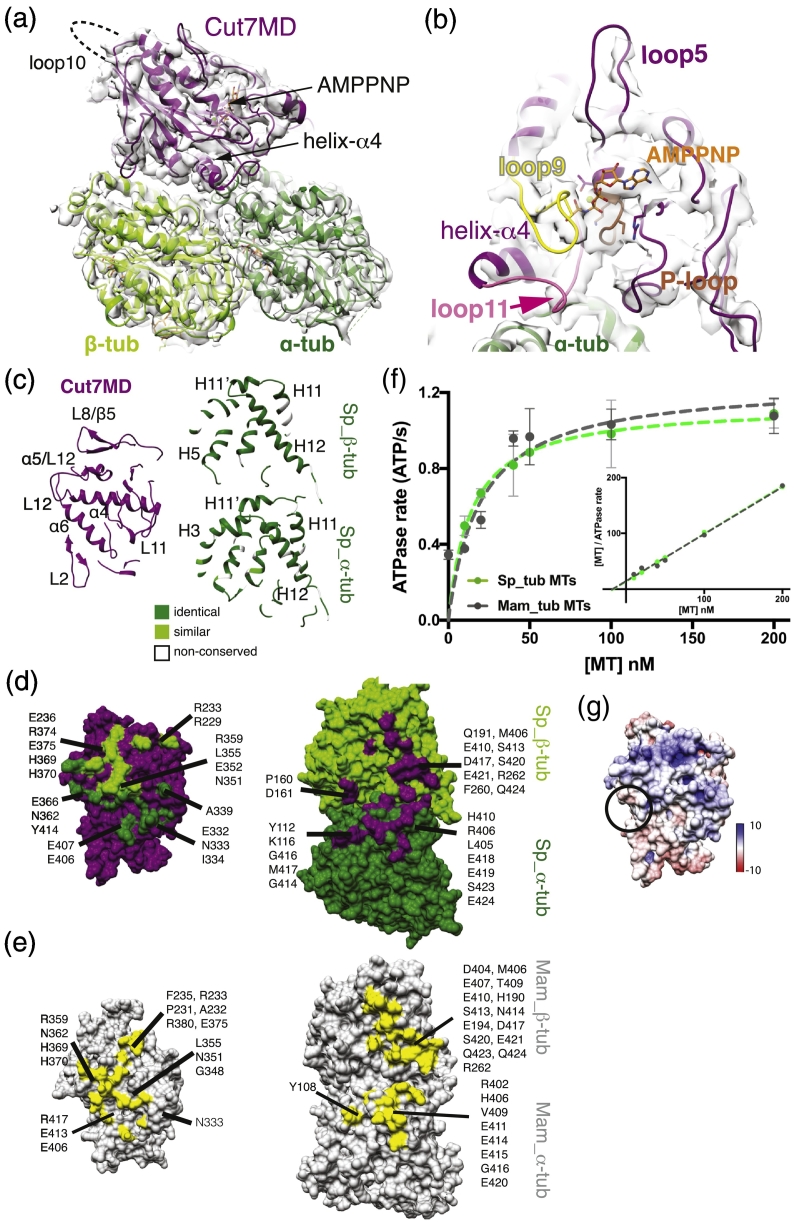
The 4.5 A resolution reconstruction of *S. pombe* Cut7MD-AMPPNP bound to Sp_tub MT reveals the distinct footprint of Cut7 on Sp_tub MTs. (a) The asymmetric unit (αβ-tubulin + Cut7MD) of the reconstruction viewed toward the nucleotide binding pocket with the Cut7MD homology model and Sp_tub atomic model docked into the density. The position of the disordered loop 10 is indicated by the dotted line. (b) View of the Cut7MD nucleotide binding pocket showing helix-α4 at the MT surface, the conserved nucleotide coordinating loops, P-loop (brown), loop9 (yellow) and loop11 (pink), as well as loop5 (purple) emerging away from the nucleotide binding site. The pink arrow indicates the separation of density corresponding to loop11 and the MT surface. (c) Ribbon depiction of longitudinal slices through the Cut7MD model (left) and Sp_tub αβ-tubulin (right) showing the structural elements involved in binding between motor and MT track. The αβ-tubulin ribbon is colored according to sequence conservation with mammalian tubulin; note, while loop2 (L2) lies close to the MT surface, there is no evidence in the EM density of a direct connection between this loop and the MT. (d) View of the MT binding surface of Cut7MD (left) and view of the Cut7MD binding surface of Sp_tub αβ-tubulin (right) with residues < 4 Å distant from each binding partner labeled and colored green and purple, respectively. This interface was evaluated using the model coordinates. (e) View of the MT binding surface of Cut7MD (left) and view of the Cut7MD binding surface of Mam_tub αβ-tubulin (right) with residues < 4 Å distant from each binding partner labeled and colored yellow, respectively. As above, this interface was evaluated using the model coordinates. (f) Cut7MD steady-state ATPase rate as a function of [MT] for Sp_tub (green) and Mam_tub (gray). Data were fit to a Michaelis–Menten kinetic yielding values for Cut7MD *V*_max_ and *K*_0.__5_MT, respectively, of 1.14 ± 0.02 ATP/s and 13.9 ± 1.1 nM on Sp_tub MTs (*R*^2^ = 0.990) and 1.25 ± 0.1 ATP/s and 19.9 ± 5.8 nM on Mam_tub MTs (*R*^2^ = 0.918), differences that are not statistically significant (*p* > 0.99). Inset, the same data presented as Hanes–Woolf plot, yielding values for Cut7MD *V*_max_ = 1.17 ± 0.06 ATP/s and *K*_0.5_MT = 13.4 ± 1.1 nM on Sp_tub MTs (*R*^2^ of fit = 0.999) and 1.19 ± 0.20 ATP/s and *K*_0.5_MT = 14.3 ± 3.4 nM on Mam_tub MTs (*R*^2^ = 0.993), which are not statistically significantly different (*p* > 0.99). (g) Cut7MD MT binding interface colored by surface charge. The approximate position of the adjacent negatively charged β-tubulin C-terminal tail is marked by a circle. A recombinant His_6_-tagged Cut7 monomeric construct, residues 67–432 lacking its N-terminal extension (Cut7MD) in a pET151D-TOPO vector (Invitrogen), was expressed in BL21*(DE3) *E. coli* cells as previously described [6]. In brief, cells were grown in LB medium, supplemented with 2% (w/vol) glucose with induction of protein expression by 0.5 IPTG at 18 °C for 5 h. Cells were resuspended in lysis buffer [50 mM Tris–HCl (pH 8.0), 400 mM NaCl, 1 mM MgCl_2_, 1 mM ATP, 5 mM 2-mercaptoethanol, 10% (vol/vol) glycerol and EDTA-free Protease Inhibitor Cocktail (Roche), 50 μM PMSF] and lysed using a French press. His_6_-tagged Cut7MD was purified from the clarified cell supernatant using nickel affinity chromatography, and the His_6_ tag was removed using TEV protease during overnight dialysis into 50 mM Tris–HCl (pH 8.0), 400 mM NaCl, 1 mM MgCl_2_, 1 mM ATP, 5 mM 2-mercaptoethanol and 10% (vol/vol) glycerol). Immediately prior to use, Cut7MD was buffer exchanged into BrB25 + [25 mM Pipes–KOH (pH 6.8), 30 mM NaCl, 0.5 mM EGTA, 5 mM MgCl_2_, 1 mM 2-mercaptoethanol, 5 mM AMPPNP] using a Vivaspin® column (Sartorius). To measure the Cut7MD ATPase activity, *S. pombe* MTs were assembled and stabilized by the addition of epothilone as for cryo-EM sample preparation, except that monomeric Mal3 was excluded from the polymerization mix. Mam_tub MTs were polymerized for 1 h at 37 °C, as previously described [20] using bovine tubulin (Cytoskeleton Inc., Denver, CO) and stabilized by the addition of 1 mM paclitaxel (Calbiochem). Cut7MD ATPase activity was measured using an enzyme-coupled assay [45] in a buffer consisting of 50 mM Tris (pH 8.0), 50 mM NaCl, 1 mM MgCl_2_, 0.5 mM phosphoenolpyruvate, 0.25 mM NADH, ~ 10 U/ml pyruvate kinase and ~ 14 U/ml lactate dehydrogenase and 5 mM ATP (all reagents from Sigma). The reactions, with each condition performed with 4–6 replicates, were initiated by the addition of Cut7MD at a final concentration of 1.5 μM. Activity was measured by the decrease in NADH absorbance at 340 nm for 10 min at 32 °C in a SpectraMax Plus 384 Microplate Reader (Molecular Devices).

Cut7MD contacts Sp_tub MTs using canonical kinesin structural elements: helix-α4 at the intradimer interface, with additional contacts formed with β-tubulin (primarily H12) by helix-α5 and β5/loop8 and with α-tubulin (H12) by helix-α6 (Fig. 2c; Fig. S2). The high degree of sequence conservation between Sp_tub and Mam_tub means that the residues on the surface of each MT are essentially identical (Fig. 2c, [15]), while the models used for comparison of the binding surface are fitted within the cryo-EM density with similar overall precision with respect to their protein backbone, despite the difference in resolutions between the reconstructions (Sp_tub: 4.5 Å; Mam_tub: 9.3 Å) [33]. However, the differences in the intrinsic architecture of *in vitro* polymerized Sp_tub compared to Mam_tub MTs [15] mean that the footprint of Cut7MD on Sp_tub MTs (Fig. 2d) is different compared with that on Mam_tub MTs (Fig. 2e), being skewed toward β-tubulin H3. In addition, the region around α_tub H11′ and β_tub H12 in Sp_tub MTs has fewer charged residues compared to Mam_tub MTs, and includes some additional contacts, involving α-L405, α-H410, β-F260 and β-R262, to the Cut7MD (N362, E366 and Y414). Thus, while the core contacts between motor and MT track are well conserved, the periphery of the motor–MT interface differs.

To evaluate the effect of this MT configuration on motor activity, we measured the steady-state ATPase activity of Cut7MD on Sp_tub MTs and Mam_tub MTs. Characteristic of kinesin-5s [1], Cut7MD has a very slow ATPase activity *in vitro*, which may be important in the context of its spindle function. However, the Cut7MD ATPase kinetic parameters on each MT type are not statistically significantly different (*V*_max_ = 1.14 ± 0.02 ATP/s on Sp_tub MTs and *V*_max_ = 1.25 ± 0.1 ATP/s on Mam_tub MTs; *K*_0.5_MT = 13.9 ± 1.1 nM on Sp_tub MTs and *K*_0.5_MT = 19.9 ± 5.8 nM on Mam_tub MTs, *p* > 0.99; Fig. 2f).

Point mutagenesis has revealed the role of specific residues at the kinesin–MT interface in stimulating kinesin ATPase and thus function (e.g., [10]). Our work provides a different view of the motor–MT interface, in which the underlying architecture of the MT alters otherwise very similar binding interfaces. Despite evidence that kinesin motor activity is sensitive to the stabilization mode of their underlying MT tracks (e.g., [25]), we find that alterations in the binding interface resulting from differences in the shape of the tubulin dimer and the lattice architecture can be accommodated with no measurable alteration in kinesin ATPase. Although the differences in Cut7MD footprint on each MT type are not evident in the ATPase activity of the monomer, they may ultimately manifest in the stepping properties of larger constructs. The highly flexible and therefore structurally invisible tubulin C-terminal tails of tubulin may also influence motor stepping. In Mam_tub, the C-terminal tails of α- and β-tubulin are variable between isoforms and subject to multiple post-translational modifications (e.g., [9]), whereas the Sp_tub has none [11]. Interestingly, the region of Cut7MD closest to the β-tubulin C-terminal tail is somewhat apolar (Fig. 2g), perhaps helping to explain the absence of apparent interaction between the motor and MT at this site. Future experiments will further explore these MT-dependent effects on the emergent property of direction reversibility and MT cross-linking activity of full-length Cut7 tetramers [6].

Distal to the nucleotide binding site of the MT-bound Cut7MD, the Cut7MD N- and C-termini coincide to form the functionally important cover neck bundle (CNB; Fig. 3a) [22], [34]. Density corresponding to the C-terminal neck linker, which connects the Cut7MD to the rest of the protein, is clearly visible docking along the length of the motor domain and directed toward the MT plus end. This docked conformation is consistent with the AMPPNP state of the motor and with canonical concepts of plus-end directed motor movement, which has been described for Cut7MD monomers [6], [35]. In this conformation, a short portion of the N-terminus lies above the docked neck linker, forming the CNB that contributes to force generation by kinesins.

**Fig. 3:**
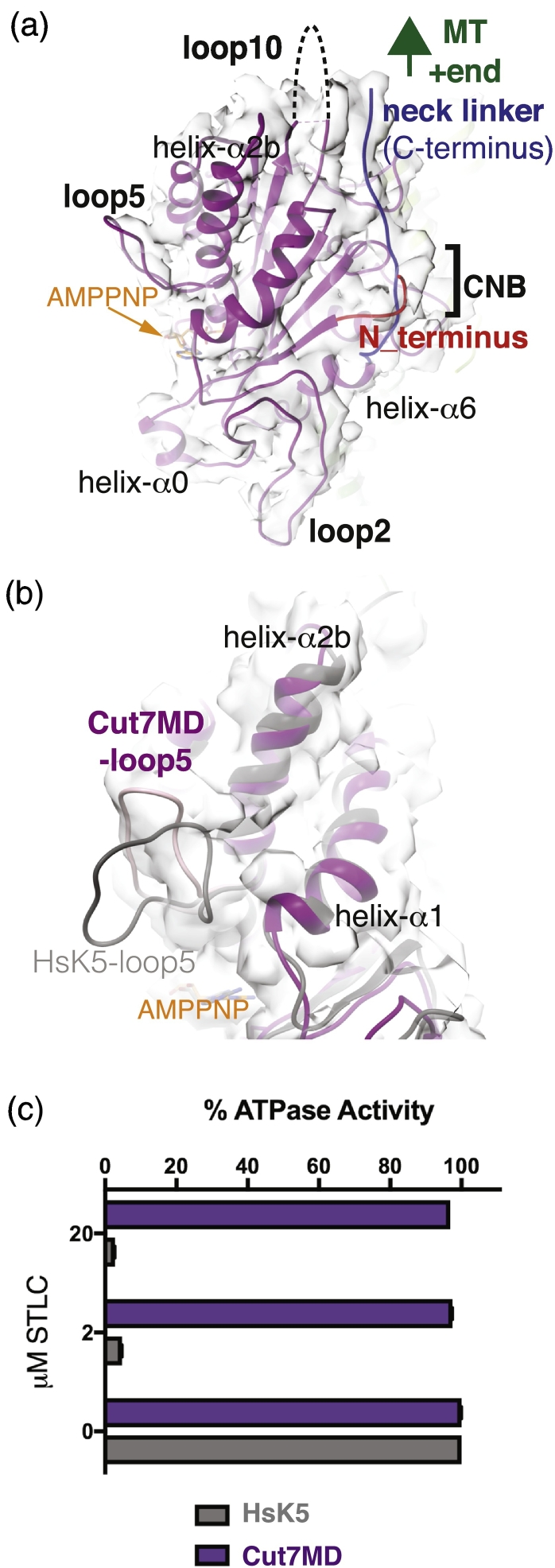
Distinctive conformation of Cut7MD and its resistance to small molecule inhibition. (a) Cut7MD CNB formation from the docked neck linker (blue)—directed toward the MT plus end—and the N-terminus (red). (b) Close-up view of the distinct conformation of Cut7MD loop5 (pink) compared to human kinesin-5 [HsK5-AMPPNP (PDB:3HQD), in gray]. (c) Steady-state MT-stimulated ATPase rates of Cut7MD (3.8 μM) and the motor domain of human kinesin-5 (1.3 μM) measured in the absence and presence of 2 μM and 20 μM of the inhibitor STLC plotted as a % of their uninhibited rates. Cut7MD is not detectably inhibited by STLC at concentrations that almost completely inhibit human kinesin-5. Mam_tub MTs (100 nM) were used in each case and 6 and 4 replicates respectively, for control and + STLC were performed. STLC was purchased from Sigma with stocks dissolved in DMSO.

At the plus end of the motor domain, there is no clear density visible corresponding to the 17-amino-acid insert in loop10 (Fig. 3a), suggesting that this loop is flexible and is thus not readily visualized. However, immediately above and protruding away from the nucleotide binding site, density corresponding to the Cut7MD-specific loop5 is clearly visible (Figs. 2b and 3b). This Cut7MD loop5 conformation is distinct from that observed for AMPPNP-bound human kinesin-5 loop5 [22], [31], as was previously observable at lower resolution [6]. The sequences of Cut7 and human kinesin-5 in this region of the motor domain are distinct (Fig. S2), and conformational sensitivity of this loop to even small changes in other kinesin-5s has been observed (Fig. 3b) [23], [36], [37], [38].

Loop5 in human kinesin-5 forms part of a binding pocket for small molecule inhibitors [1], and in addition to conformational divergence, several residues in human kinesin-5 that mediate inhibitor binding (e.g., W127, Y211) are not conserved in Cut7MD (Fig. S2). Thus, our structure is consistent with the prediction that Cut7MD would be insensitive to human-specific kinesin-5 inhibitors. We tested this idea and found that, indeed, the ATPase activity of Cut7MD is not inhibited by concentrations of the inhibitor (+)-*S*-trityl-l-cysteine (STLC) that completely block human kinesin-5 (Fig. 3c). This specificity of STLC inhibition is consistent with previous inhibitor studies using engineered proteins [37]. It also highlights that, in the future, small molecules designed to bind to this region of fungal kinesin-5s might be expected to be interesting and specific candidates for fungicides.

In summary, our study sheds new light on diversity of structure and interaction between apparently well-conserved binding partners in the cytoskeleton and highlights the importance of studying cognate motor-track combinations to elucidate physiologically relevant properties. These data describing monomeric Cut7 will provide important constraints for future models of regulated bidirectional stepping performed by the full-length motor.

## Accession numbers

The cryo-EM reconstruction that supports the findings of this study has been deposited in the Electron Microscopy Data Bank, accession number 3527. The docked coordinates reported in this paper have been deposited in the Protein Data Bank, www.pdb.org, accession number 5MLV.

## Appendix A Supplementary data

Supplementary material Image 1
