# Corrigendum to “Cryo-EM Structure (4.5 Å) of Yeast Kinesin-5–Microtubule Complex Reveals a Distinct Binding Footprint and Mechanism of Drug Resistance” [J. Mol. Biol. 431 (2019) 864-872] https://doi.org/10.1016/j.jmb.2019.01.011

**DOI:** 10.1016/j.jmb.2019.08.015

**Published:** 2020-01-17

**Authors:** Ottilie von Loeffelholz, Alejandro Peña, Douglas Robert Drummond, Robert Cross, Carolyn Ann Moores

**Affiliations:** 1 Institute of Structural and Molecular Biology, Birkbeck College, London WC1E 7HX, UK; 2 Division of Biomedical Cell Biology, Warwick Medical School, Coventry CV4 7AL, UK; 3 Centre for Integrative Biology, Department of Integrated Structural Biology, Institute of Genetics and of Molecular and Cellular Biology, 1 rue Laurent Fries, 67404 Illkirch, France; 4 Centre for Promotion of International Education and Research, Faculty of Agriculture, Kyushu University, Fukuoka 812-8581, Japan

Following release of our structures, we found that the epothilone coordinates were incorrectly docked with respect to tubulin. This is now corrected in a new deposition in the Protein Data Bank, www.pdb.org, accession number 6S8M.

The authors would like to apologize for any inconvenience caused.

