# Supplementary material for: Cryo-EM Structure (4.5-Å) of Yeast Kinesin-5–Microtubule Complex Reveals a Distinct Binding Footprint and Mechanism of Drug Resistance
Source: J Mol Biol. 2019 Feb 15;431(4):864–72. doi: 10.1016/j.jmb.2019.01.011 (PMC6378684; doi:10.1016/j.jmb.2019.01.011)
Supplement: Supplementary file 1 — Supplementary material [file mmc1.pdf]

4.5 Å cryo-EM structure of yeast kinesin-5-microtubule complex reveals a distinct binding footprint and mechanism of drug resistance

Supplementary Material

Ottilie von Loeffelholz[1,3], Alejandro Peña[1], Douglas Robert Drummond[2,4], Robert Cross[2], Carolyn Ann Moores[1*]

[1] Institute of Structural and Molecular Biology, Birkbeck College, London WC1E 7HX, U.K.
[2] Division of Biomedical Cell Biology, Warwick Medical School, Coventry, CV4 7AL, U.K.
[3] Present address: Centre for Integrative Biology, Department of Integrated Structural Biology, Institute of Genetics and of Molecular and Cellular Biology, 1 rue Laurent Fries, 67404 Illkirch, France
[4] Present address: Centre for Promotion of International Education and Research, Faculty of Agriculture, Kyushu University, Fukuoka 812-8581, Japan.

* Corresponding Author/Lead Contact
Carolyn A. Moores; ORCID ID: 0000-0001-5686-6290
E-mail: c.moores@mail.cryst.bbk.ac.uk

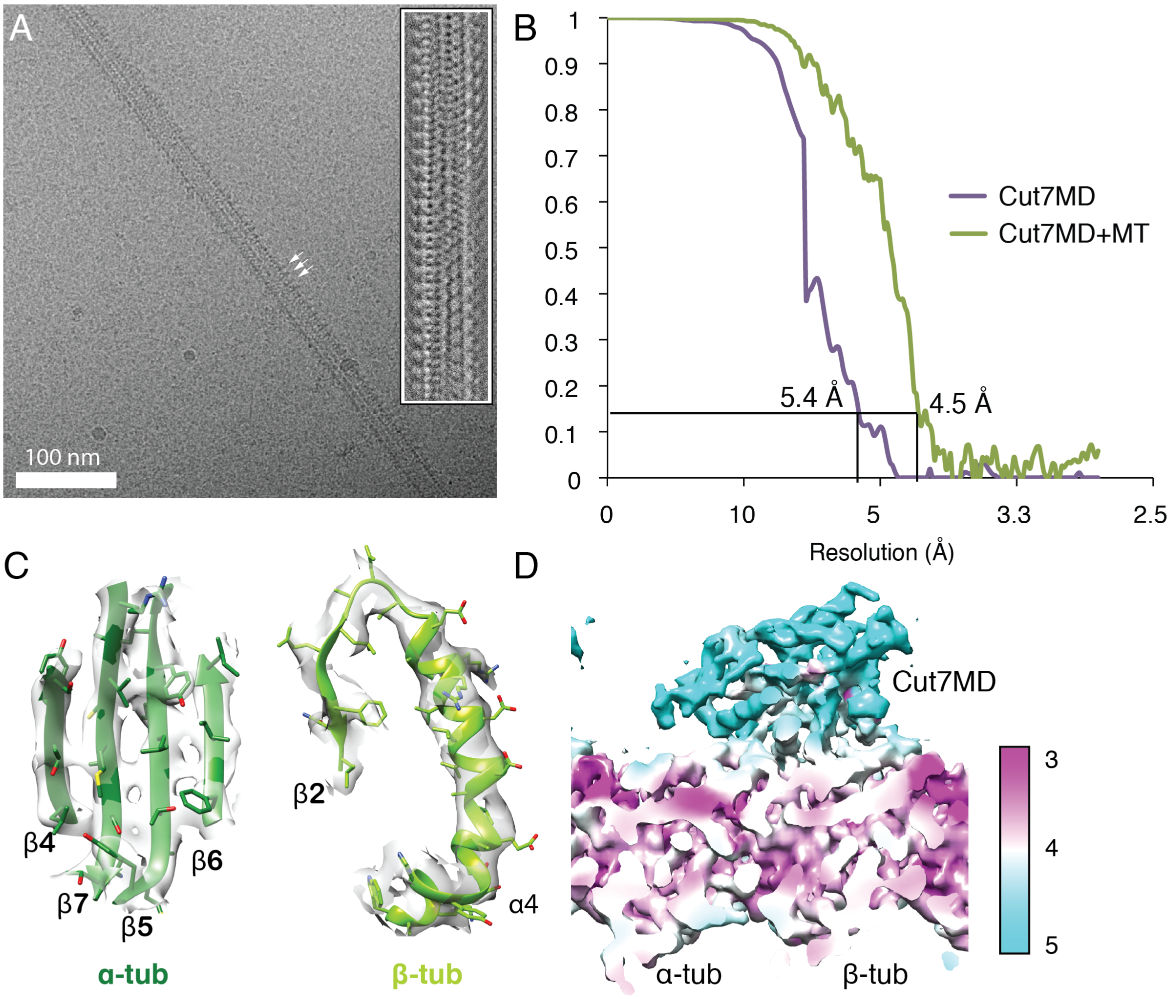

Figure S1. Visualization of Cut7MD binding to Sp_tub MTs and evaluation of the Cut7MD-Sp_tub MT cryo-EM reconstruction resolution. (A) Cryo-EM micrograph of Cut7MD-decorated Sp_tub MTs showing canonical binding of the motor domain every 8nm on the MT lattice (arrows); inset, MT segment average also shows clear Cut7MD binding every 8nm, as well as the characteristic moiré repeat of the Sp_tub 13PF MTs; (B) FSC curves for the overall reconstruction (green) estimated to be 4.5 Å by the 0.143 criterion and for the Cut7MD region specifically (purple); (C) Visualization of high resolution features in the Sp_tub density supports the near-atomic resolution quality of the reconstruction; (D) Depiction of local resolution estimate in the reconstruction using the *blocres* program implemented in Bsoft (Cardone et al., 2013) indicates the presence of a resolution gradient between the MT and kinesin, as is typical in motor-MT complexes (Kellogg et al., 2017); the average resolution in the tubulin region of the reconstruction is ~ 4.4 Å with some regions substantially better than this, while resolution of the Cut7MD is ~ 5 Å. Cut7MD and MT regions of the reconstruction were independently sharpened. Guided by the local resolution, we focused on the interpretation of secondary structure information only for Cut7MD, while the higher resolution in the MT part and the MT-Cut7MD interface allowed interpretation of possible side-chain interactions.

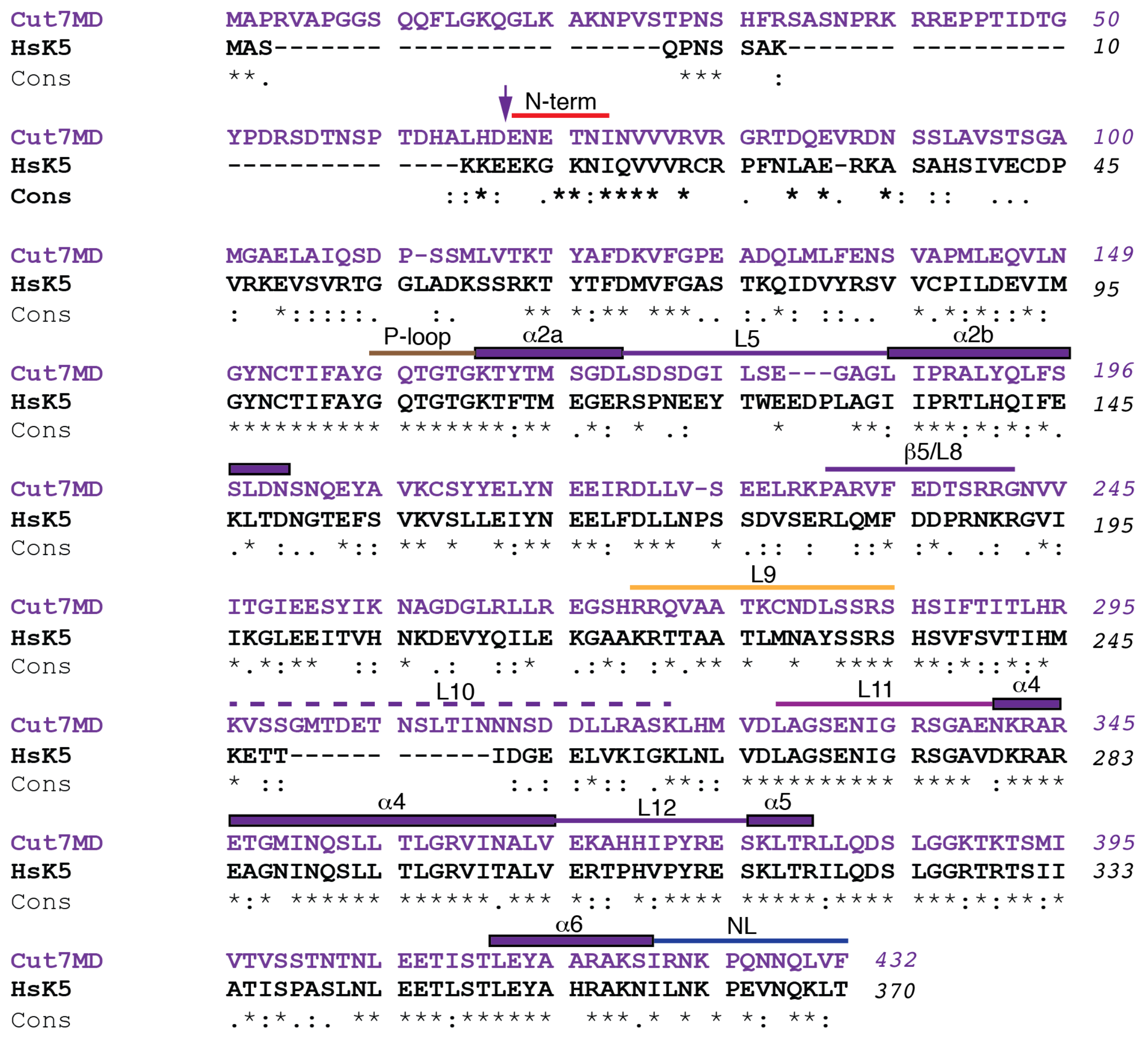

Figure S2. Sequence alignment of the Cut7 (Uniprot: P24339) and human Kif11 (Uniprot: P52732) motor domain sequences. Motor domain sequences were aligned using T-Coffee (Notredame et al., 2000) with sequence conservation depicted below the alignment. Secondary structure elements referenced in the text are annotated and the N-terminus of the Cut7MD construct used in this study is indicated with an arrow.

References

Kellogg, E.H., Hejab, N.M.A., Howes, S., Northcote, P., Miller, J.H., Diaz, J.F., Downing, K.H., and Nogales, E. (2017). Insights into the Distinct Mechanisms of Action of Taxane and Non-Taxane Microtubule Stabilizers from Cryo-EM Structures. J Mol Biol *429*, 633-646.

Notredame, C., Higgins, D.G., and Heringa, J. (2000). T-Coffee: A novel method for fast and accurate multiple sequence alignment. J Mol Biol *302*, 205-217.
